# Across ancestries, HLA-B∗08:01∼DRB1∗03:01 (DR3) and HLA-DQA∗01:02 (DR2) increase the risk to develop juvenile-onset systemic lupus erythematosus through low complement C4 levels

**DOI:** 10.1016/j.jtauto.2025.100268

**Published:** 2025-01-07

**Authors:** Yves Renaudineau, Amandine Charras, Valentina Natoli, Nicolas Congy-Jolivet, Sam Haldenby, Xuan Liu, Yongxiang Fang, Eve MD. Smith, Michael W. Beresford, Christian M. Hedrich, UK jSLE Cohort Study Group, Carla Roberts, Eslam Al-Abadi, Kate Armon, Kathryn Bailey, Coziana Ciurtin, Janet Gardner-Medwin, Kirsty Haslam, Daniel P. Hawley, Alice Leahy, Valentina Leone, Flora McErlane, Gita Modgil, Clarissa Pilkington, Athimalaipet V. Ramanan, Satyapal Rangaraj, Phil Riley, Arani Sridhar

**Affiliations:** i Department of Women's & Children's Health, Institute of Life Course and Medical Sciences, University of Liverpool, United Kingdom; j Department of Rheumatology, Birmingham Children's Hospital, Birmingham, United Kingdom; k Department of Paediatric Rheumatology, Cambridge University Hospitals, Cambridge, United Kingdom; l Department of Paediatric Rheumatology, Oxford University Hospitals NHS Foundation Trust, Oxford, United Kingdom; m Centre for Adolescent Rheumatology, University College London, London, United Kingdom; n Department of Child Health, University of Glasgow, Glasgow, United Kingdom; o Department of Paediatrics, Bradford Royal Infirmary, Bradford, United Kingdom; p Department of Paediatric Rheumatology, Sheffield Children's Hospital, Sheffield, United Kingdom; q Department of Paediatric Rheumatology, Southampton General Hospital, Southampton, United Kingdom; r Department of Paediatric Rheumatology, Leeds Children Hospital, Leeds, United Kingdom; s Paediatric Rheumatology, Great North Children's Hospital, Royal Victoria Infirmary, Institute of Cellular Medicine, Newcastle University, Newcastle upon Tyne, United Kingdom; t Department of Paediatrics, Musgrove Park Hospital, Taunton, United Kingdom; u Department of Paediatric Rheumatology, Great Ormond Street Hospital, London, United Kingdom; v University Hospitals Bristol NHS Foundation Trust & Bristol Medical School, University of Bristol, Bristol, United Kingdom; w Department of Paediatric Rheumatology, Nottingham University Hospitals, Nottingham, United Kingdom; x Department of Paediatric Rheumatology, Royal Manchester Children's Hospital, Manchester, United Kingdom; y Department of Paediatrics, Leicester Royal Infirmary, Leicester, United Kingdom; a Immunology Department Laboratory, Referral Medical Biology Laboratory, Institut Fédératif de Biologie, Toulouse University Hospital Centre, France; b INFINITy, Toulouse Institute for Infectious and Inflammatory Diseases, INSERM U1291, CNRS U5051, University Toulouse III, Toulouse, France; c Department of Women's & Children's Health, Institute of Life Course and Medical Sciences, University of Liverpool, United Kingdom; d Dipartimento di Neuroscienze, Riabilitazione, Oftalmologia, Genetica e Scienze Materno-Infantili, Università degli Studi di Genova, Genoa, Italy; e UOC Reumatologia e Malattie Autoinfiammatorie, IRCCS Istituto Giannina Gaslini, Genoa, Italy; f CRCT, INSERM, UMR, 1037, University Toulouse III, Toulouse, France; g Centre for Genomic Research, Shared Research Facilities, University of Liverpool, United Kingdom; h Department of Rheumatology, Alder Hey Children's NHS Foundation Trust, Liverpool, United Kingdom

**Keywords:** Lupus, Genetic, Juvenile-onset, SLE, HLA, Haplotype, Disease activity, Complement, C4

## Abstract

**Objective:**

Systemic lupus erythematosus (SLE) is a systemic autoimmune/inflammatory disease with a strong genetic component. Genetic burden is higher in children when compared to patients with adult-onset SLE, contributing to earlier disease expression and more severe phenotypes. The human leukocyte antigen (HLA) cluster on chromosome 6p21.3 is among the most variable genomic regions, representing a major risk-factor for SLE in adults. Its impact on juvenile-onset (j)SLE remains largely unstudied.

**Methods:**

High-resolution sequencing of HLA class I (A, B, C), class II (DRB1, DQA1, DQB1) and class III (complement *C2*) was undertaken in the multi-ancestral UK JSLE Cohort including participants of Caucasian (n = 151, 48.8 %), Asian (n = 108, 35.0 %) and African/Caribbean (n = 50, 16.2 %) descent. Considering ancestral variation, clinical associations were tested at the level of alleles (2-field resolution), associated HLA protein sequences (antigen binding domains, 4-field resolution), and extended haplotypes (DRh).

**Results:**

Although important ancestral recombination was reported for HLA-DR2 and -DR3 haplotypes, risk associated with jSLE was conserved at related alleles (DR2h: DRB1∗15:01, DQA∗01:02, DQB1∗06:02; DR3h: C∗07:02 [Asian], B∗08:01, *C2* rs9332730 [Asian], DRB1∗03:01). HLA-DR7 haplotypes (DRB1∗07:01, OR = 0.44, 95 % CI:0.27–0.72, p = 0.0004; DQA1∗02:01, OR = 0.34, 95 % CI:0.21–0.56, p = 1.8 × 10^−6^) protect Asians from jSLE development. Among 23 clinical variables recorded, the main association was found between low levels of complement C4 in Caucasian carriers of HLA-DR3h. This was not the case in Asians due to recombination with HLA-C∗07:02 and integration of the *C2* rs9332730 minor allele. Low C4 serum levels associated with HLA-DQA1∗01:02 (DR2h) in Caucasians after excluding HLA-DR3h carriers from the analysis. An association between low white blood cell counts and HLA-A∗03:01P was observed across ancestries.

**Conclusion:**

Genetic variation in the *HLA* cluster associates with organ domain involvement (hematological) and complement levels in jSLE. Lupus-associated *HLA* haplotypes vary between ancestral groups, underscoring the importance of multi-ancestral approaches to genetic studies in SLE and other autoimmune/inflammatory diseases.

## Highlights

• Lupus-associated HLA haplotypes vary between ancestral groups.
• Across ancestries, HLA-DR2 and -DR3 haplotypes act as jSLE risk factors.
• In Asians, HLA-DR7 haplotypes protect from jSLE development.
• In jSLE, low serum C4 levels may be the result of ancestral haplotypes.

## 1 Introduction

Systemic lupus erythematosus (SLE) is a systemic autoimmune/inflammatory disease with a strong genetic component [1,2]. Approximately 15–20 % of SLE patients develop disease prior to their 18th birthday. Disease onset during childhood and/or adolescence, when compared to patients with adult-onset SLE, associates with more severe clinical phenotypes, the need for more aggressive treatment, and less favorable outcomes [3,4]. Furthermore, across all age groups, SLE is more common and severe in people of non-European ancestry [1,[5], [6], [7]]. Because jSLE frequently affects multiple organ systems within an individual patient and its significant overlap with other autoimmune/inflammatory diseases (such as idiopathic arthritis, scleroderma, Sjogren's syndrome, etc.), it is a prime example for the concept of “poly-autoimmunity”, the presence of more than one well-defined autoimmune disease in a single patient [8,9]. Thus, understanding SLE may contribute to a better understanding also of these associated autoimmune/inflammatory diseases.

A combination of pathomechanistic factors contribute to the development of SLE, including genetic and epigenetic alterations, immune dysfunction, and environmental impact [2,10,11]. However, the contribution of these factors varies between individual patients, ancestries and age groups. Early disease onset and associated clinical severity have been attributed to an increased genetic burden in juvenile-onset (j)SLE when compared to patients with disease onset during adulthood [1,7,12]. A relatively small proportion of SLE patients carry gene variants with high effect size that can cause “monogenic SLE” or SLE-like disease. Genes linked to “monogenic SLE” are most often involved in the classical complement pathway, Toll-like receptor/interferon signaling, and lymphocyte activation/function [7,13,14]. The majority of SLE patients exhibit a combination of gene variants, so-called risk alleles, that increase the risk for disease development but are not “strong” enough *per se* to cause lupus [1,2,6,7,15]. Among these risk alleles are variants affecting the four mega-base spanning *Human Leukocyte Antigen* (*HLA*) super-locus on chromosome 6p21.3 [16]. The study of this highly variable region of the human genome is complicated by its gene density with 253 gene loci, including 132 coding genes, involved in innate and acquired immune responses, and the association of genes in conserved haplotypes [16]. While early studies focused on highly polymorphic HLA class I and class II genes (including receptors that present peptides to CD8 [class I] or CD4 [class II] T cells), more recent analyses also considered associations with non-*HLA* genes within the cluster (e.g., complement *C2*, *C4A* and *C4B*) [17]. Notably, in SLE, more *HLA* risk alleles are present when compared to other immune-mediated diseases [18], but their analysis is complicated by inter-ancestral variation. For instance, HLA-DR2 (DRB1∗1501/DQB1∗0602) and -DR3 (DRB1∗0301/DQB1∗0201; MIM∗142860) haplotypes that are associated with SLE in Caucasian but not in Asian or African ancestries [19].

Associations between SLE and genetic variation of the *HLA* cluster have almost exclusively been investigated in adult-onset SLE patients. This study, in contrast, has investigated a large multi-ancestral cohort of jSLE patients. A total of 309 jSLE patients enrolled in the UK JSLE Cohort Study and Repository were included. Diversity of HLA class I (A, B, C), class II (DRB1, DQA1, and DQB1) and class III (complement C2) clusters were analysed at the allele, protein, and extended haplotype level; variation was associated with clinical phenotypes.

## 2 Materials and methods

### 2.1 Study cohort

A total of 309 children and young people with SLE enrolled in the UK JSLE Cohort Study (http://www.liv.ac.uk/ukjsle) were included in this study. Patients classified as having “monogenic SLE” and those with “mixed ancestry” were not included here [14,15,20].

The following clinical datasets were collected: (i) demographic information (age at disease onset, sex, ancestry: self-reported Caucasian, Asian, African/Caribbean descent; participants with mixed ancestry were excluded from this study); (ii) global disease activity scores, including the paediatric British Isles Lupus Assessment Grade 2004 (pBILAG-2004) and the SLE Disease Activity Index 2000 (SLEDAI-2K) score [21,22]; (iii) severity (using the numeric version of the pBILAG-2004 score: A = 12, B = 8, C = 1, and D/E = 0) of disease at the organ/system level [23]; and (iv) SLE-associated laboratory parameters, including anti-double-stranded (ds)DNA antibodies, anti-extractable nuclear (ENA) antibodies, complement C3 and C4 levels, and white blood cell (WBC) count as previously described [24,25].

Conducted in accordance with the declaration of Helsinki, the study received ethical approval from the National Research Ethics Service Northwest (REC 06/Q1502/77), and all participants and/or their legal guardians gave written informed patient assent/consent.

### 2.2 Sequencing and bioinformatic approach

Sequence capture probes were designed to target *HLA-A, HLA-B, HLA-C, HLA-DRA, HLA-DRB1, HLA-DQA1, HLA-DQB1, HLA-DQB2, HLA-DPA1, HLA-DPB1* and *C2* gene loci. Sequencing libraries were prepared from genomic DNA, hybridized to the probes (NimbleGen/Roche, Basel, Switzerland) and sequenced with 150bp paired-end reads using Illumina MiSeq technology (Illumina, Inc., San Diego, USA). Demultiplexing, adaptor and quality trimming (Cutadapt v1.2.1, Sickle v1.2) of reads was performed [Cutadapt v1.2.1 (https://doi.org/10.14806/ej.17.1.200), Sickle v1.2 (https://github.com/najoshi/sickle)]. Trimmed reads were aligned to the human reference genome (GRCh38, including ALT alleles and decoy sequences) and processed with BWAkit (v0.7.15) (https://doi.org/10.6084/M9.FIGSHARE.963153.V1), to genotype HLA alleles. The top matching allele, or allelic pairs in cases of heterozygosity, was identified and reported for each locus. Non-HLA sequencing data were aligned against the human reference genome (hg38) using BWA [26], and variants were subsequently detected with *Genome Analysis Toolkit Software* (GATK) [26,27]. GATK base quality score recalibration was applied, variants were filtered using the GATK Variant Filtration tool and annotated using SnpEFF for the complement *C2* gene [28].

HLA allele frequencies at 2-field resolution in the UK (White British, n = 475), Asia (n = 6970), and Africa (n = 4985) were obtained from the Allele Frequency Net Database (AFND, www.allelefrequencies.net) [29], and the protein (P) designation was attributed to HLA sequences at 4-field resolution having the same antigen binding domain according to the HLA alleles nomenclature website (https://hla.alleles.org) [[30], [31], [32]].

Minor allele frequencies (MAF) in UK controls (White British, n = 276,338) were obtained from the UK blood bank database (https://decaf.decode.com/) [33]; MAF in Asian (n = 48,640) and African (n = 15,100) controls were retrieved from the *genome Aggregation Database* (gnomAD) v2.1.1 (https://gnomad.broadinstitute.org/) [34].

### 2.3 Statistical analysis

Quantitative data are presented as median ± interquartile ranges (IQ) and analysed using ANOVA non-parametric tests with Dunn's test applied for post-hoc multiple comparisons in analysis of variance (ANOVA) when necessary. For categorical data, differences among groups were analysed using the Fisher's exact test. Principal component analysis (PCA) was performed to test ancestry associations between haplotypes using the ClustVis webtool (http://biit.cs.ut.ee/clustvis/) [35]. For quantitative trait loci (QTL) analysis, Spearman's correlation test was performed using allele/protein/haplotype numbers (0, 1, and 2). The Benjamini-Hochberg False discovery rate p value (pFDR) was selected to control p-values for multiple testing, where appropriate. Results were visualised using PRISM10.3 (GraphPad Software, La Jolla, CA, USA).

## 3 Results

### 3.1 Cohort

The majority of participants included in this study, 259/309 (83.8 %), were female. Three main ancestral groups were represented, Caucasians (n = 151, 48.8 %) with a predominance of British origin (127/151, 80.1 %), Asians (n = 108, 35.0 %), and participants of African/Caribbean ancestry (n = 50, 16.2 %). When compared to Caucasians, minor demographic differences were observed in jSLE patients of Asian (sex distribition; *p* = 0.03) or African/Caribbean ancestry (lower age at diagnosis/1st visit; lower global/mucocutaneous disease activity (0.003<*p* < 0.05) (Table 1). Minor differences in demographic and disease characteristics from this group to previous reports are primarily related to the exclusion of patients with “monogenic jSLE” [14,15,20].

**Table 1:** Demographic and clinical characteristics.

Characteristics	Caucasians (n = 151)	Asian ancestry (n = 108)	African/Caribbean ancestry (n = 50)	p values
Female: Male	133:18	82:26∗	44:6	**0.027**
Age at diagnosis (years)	13.0 (10.7–14.7)	12.9 (10.6–14.6)	10.7 (8.9–13.3)∗	**0.011**
Age at 1st visit	13 (11–15)	14 (11–16)	12.0 (9.2–14.0)∗	**0.044**
pBILAG-2004	8 (3–15)	9 (4–17)	5 (1–12)∗	**0.044**
SLEDAI-2K	10 (4–17)	8 (4–14)	5 (1–12)∗	**0.003**
Constitutional^&^	0 (0–8) [38 %]	0 (0–8) [47 %]	0 (0–3) [38 %]	0.171
Mucocutaneous^&^	1 (0–8) [62 %]	1 (0–8) [66 %]	0 (0–2.8) [36 %]∗	**0.002**
Musculoskeletal^&^	1 (0–8) [60 %]	1 (0–1) [52 %]	0 (0–1) [44 %]	0.057
Haematological^&^	1 (0–8) [63 %]	1 (0–8) [63 %]	1 (0–8) [68 %]	0.773
Renal^&^	0 (0–8) [40 %]	0 (0–8) [36 %]	0 (0–8) [36 %]	0.902
Neurological^&^	0 (0-0) [8 %]	0 (0-0) [12 %]	0 (0-0) [12 %]	0.500
Cardiorespiratory^&^	0 (0-0) [15 %]	0 (0-0) [11 %]	0 (0-0) [6 %]	0.230
Gastrointestinal^&^	0 (0-0) [7 %]	0 (0-0) [9 %]	0 (0-0) [4 %]	0.475
Ophthalmic^&^	0 (0-0) [1 %]	0 (0-0) [2 %]	0 (0-0) [2 %]	0.920
Anti-dsDNA IgG, IU/mL [%, <10 IU/mL]	37 (5–196) [56 %]	28 (7–132) [56 %]	38 (11–141) [56 %]	0.867
C3 level, g/L [%, <0.88 g/L]	0.94 (0.61–1.22) [42 %]	0.96 (0.58–1.29) [42 %]	0.92 (0.69–1.15) [47 %]	0.964
C4 level, g/L [%, <0.16 g/L]	0.11 (0.06–19) [66 %]	0.15 (0.08–0.26) ∗ [51 %]	0.08 (0.14–0.2) [57 %]	0.024
WBC (10^9^/L)	5.9 (4.0–8.9)	4.9 (4.0–7.4)	5.9 (4.1–8.6)	0.115

A total of 309 participants with juvenile-onset systemic lupus erythematosus (jSLE) were included in this study. Presented are demographic and clinical features at their 1st study visit, stratified by ancestral group. Quantitative results are presented as median ± first-third quartile. Significant Dunn's multiple comparison test factor (∗); pBILAG organ domain involvement (^&^); pBILAG-2004 score: A = 12, B = 8, C = 1, and D/E = 0 (for each organ domain).

### 3.2 Multi-ethnic allele association analysis

Using a high resolution 2-field approach for HLA class I/II, among all participants, a total of 20 HLA-A∗, 20 HLA-C∗, 24 HLA-B∗, 17 HLA-DRB1∗, 14 HLA-DQA1∗, and 15 HLADQB1∗ alleles were identified at a frequency >1 %. Furthermore, there were 7 SNPs affecting the complement *C2* gene (Chr6: 31897847–31944701, GRCh38) with a minor allele frequency (MAF) > 1 %.

**Fig. 1:**
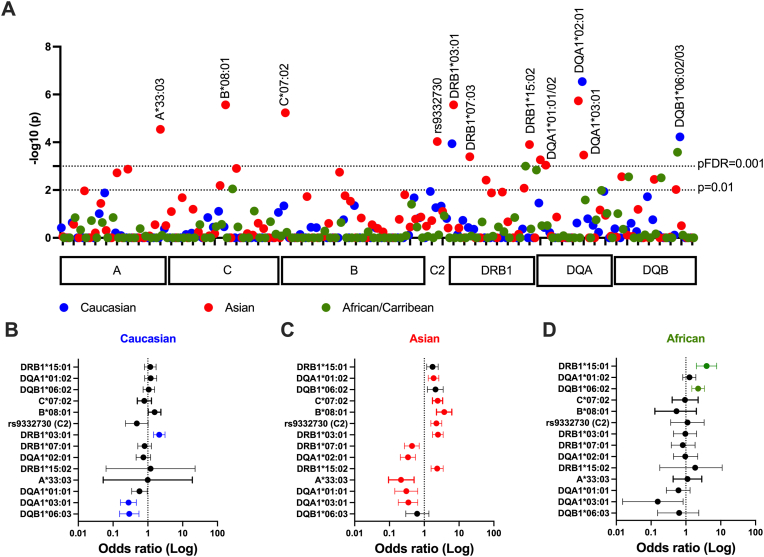
**Ancestry-differential HLA allele diversity in jSLE**. A: Manhattan plot according to ancestral groups: Caucasians in blue, Asians in red, African/Caribbean descendants in green. Ancestry-related controls were obtained from several databases (Materials and Methods). HLA-class I, HLA-class III (C2 gene), and HLA-class II regions are represented on the horizontal axis with – Log10 (p values) on the vertical axis. The statistical threshold was fixed at pFDR = 0.001; and the p = 0.01 cut-off is presented (doted lines). **B-D:** Odds ratios of HLA alleles significantly associated with jSLE across ethnicities. Significant risk and protective factors are displayed in colour (pFDR<0.001).

Considering a pFDR<0.001 as a cutoff, we identified several ancestry differential SLE-associated haplotypes (h), including DR2h and DR3h, and protective alleles in HLA DR7h (Fig. 1):

- HLA-DR2h associations included HLA-DRB1∗15:01 (African/Caribbean: OR = 3.9, 95 % CI:2.0–7.69, p = 0.00099), HLA-DQA1∗01:02 (Asian: OR = 1.86, 95 % CI:1.3–2.6, p = 0.0009), and HLA-DQB1∗06:02 (Africa/Caribbean: OR = 2.25, 95 % CI:1.48–3.4, p = 0.0003).
- HLA-DR3h associations included HLA-B∗08:01 (Asian: OR = 3.75, 95 % CI:2.25–6.21, p = 5 × 10^−6^), and HLA-DRB1∗03:01 (Caucasian: OR = 2.1, 95 % CI:1.46–3.11, p = 0.0001; and Asian: OR = 2.5, 95 % CI:1.73–3.5, p = 2.7 × 10^−6^).
- Protective associations in HLA-DR7h included HLA-DRB1∗07:01 (Asian: OR = 0.44, 95 % CI:0.27–0.72, p = 0.0004) and HLA-DQA1∗02:01 (Asian: OR = 0.34, 95 % CI:0.21–0.56, p = 1.8 × 10^−6^).

Additional, not previously reported risk associations were observed in jSLE patients of Asian descent, including HLA-C7∗07:02 (OR = 2.7, 95 % CI:1.73–3.39, p = 2.7 × 10^−6^), DRB1∗15:02 (OR = 2.35, 95 % CI:1.57–3.49, p = 0.0001), and the *C2* rs9332730 splice variant (OR = 2.23, 95 % CI:1.55–3.19, p = 9.3 × 10^−5^). Further protective associations were retrieved for HLA-A∗33:03 (Asian: OR = 0.21, 95 % CI:0.09–0.51, p = 2.8 × 10^−5^), HLA-DQA1∗01:01 (Asia: OR = 0.31, 95 % CI:0.14–0.65, p = 0.0005), HLA-DQA1∗03:01 (Caucasian: OR = 0.28, 95 % CI:0.16–0.46, p = 3 × 10^−7^; Asian: OR = 0.35, 95 % IC: 0.18–0.65, p = 0.0003), and HLA-DQB1∗06:03 (Caucasian: OR = 0.29, 95 % CI:0.15–0.56, p = 6 × 10^−5^) (Fig. 1B and C & D).

Overall, 14 associations differentially affecting ancestries were reported across the *HLA* super-locus, which may result in part from distinct haplotype recombination (Fig. 1).

### 3.3 Ancestry-related haplotype diversity in jSLE

**Fig. 2:**
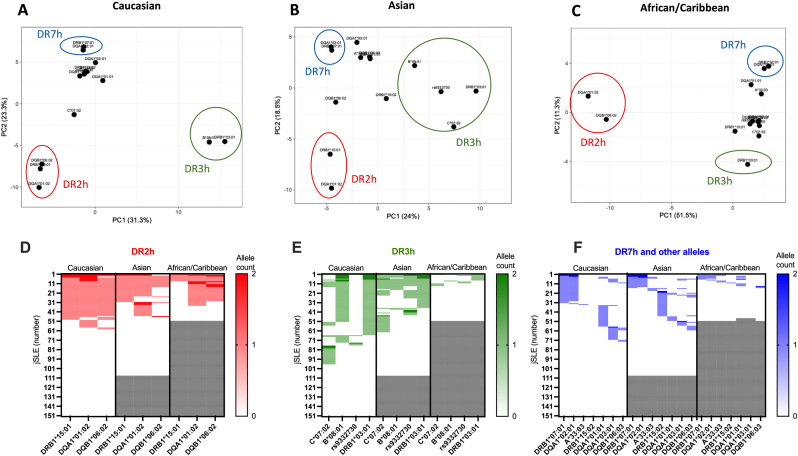
**HLA-DR2(h), -DR3h, and -DR7h haplotypes differentially associate with jSLE across ancestral groups. A-C**: Principal component analysis (PCA) based on 14 risk/protective alleles identified in jSLE patients (Fig. 1) of Caucasian (**A**), Asian (**B**) or African/Caribbean ancestry (**C**). **D:** HLA-DR2h, **E:** HLA-DR3h and **F:** HLA-DR7h and other alleles according to the ancestry group.

To further characterize jSLE-associated HLA haplotypes across ancestries, unsupervised PCA was performed. Three haplotypes were mainly associated with jSLE in Caucasian participants: HLA-DR2h (HLA-DRB1∗15:01∼DQA1∗01:02∼DQB1∗06:02, affecting 33.8 %), HLA-DR3h (HLA-B∗08:02∼DRB1∗03:01, affecting 30.4 %), and HLA-DR7h (HLA-DRB1∗07:01∼DQA1∗02:01, affecting 19.2 %) (Fig. 2A).

Ancestral variation was observed for HLA-DR2h and HLA-DR3h (Fig. 2B–F). Within the HLA-DR2h, HLA-DQA∗01:02 was associated with jSLE across ancestries, while weaker associations were seen for HLA-DQB1∗06:02 in Asian and HLA-DRB1∗15:01 in African/Caribbean patients (Fig. 2D). HLA-DRB1∗03:01 and HLA-DR3h were only recorded in jSLE patients of African/Caribbean ancestry (Fig. 2E). Alternative HLA-DR3h isoforms characterized Asian jSLE patients (HLA-C∗07:02∼HLA-B∗08:02∼rs9332730∼DRB1∗03:01) that resulted from the integration of HLA-C∗07:02 (instead of HLA-C∗07:01 classically reported in the ancestral HLA-DR3h) and the presence of the *C2* rs9332730 splice variant.

### 3.4 HLA associations with clinical presentation

Previous reports suggested genetic variation of the HLA-super locus to contribute to SLE pathophysiology and phenotypic diversity in adult-onset patients [[36], [37], [38]]. To explore this hypothesis in jSLE, quantitative trait loci (QTL) analysis was conducted to test associations between clinical parameters and: i) the here identified 14 SLE-associated risk/protective HLA alleles, and HLA alleles with a population frequency >5 % (n = 37); ii) resulting HLA proteins to explore effects mediated by disease driving epitopes (n = 30); and iii) extended HLA haplotypes, including ancestral variants, to test for the presence of non-HLA allele risk factors in linkage disequilibrium (n = 7).

**Fig. 3:**
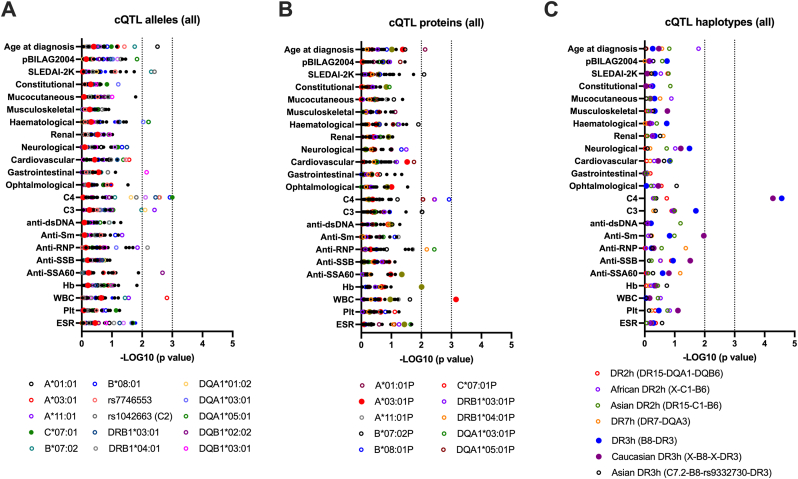
***jSLE-associated HLA quantitative trait loci (QTL). A:****Manhattan plot of HLA alleles (x A∗, x C∗, B∗, x C2-factor B, x DRB1∗, x DQA1∗, and xDQB1∗) with a frequency ≥0.05 and their association with age at diagnosis (years), disease activity (pBILAG 2004, SLEDAI-2K), disease activity (pBILAG 2004 organ domains), and clinical features.****B:****Concordance with HLA protein sequence.****C:****Concordance with haplotypes DR2(h), DR3h, DR7h and ancestral variants. On the y axis,–Log10(p values) are presented, assessing concordance between allele/protein number (0, 1, and 2) or haplotype (DR3h/DR7h: 0, 2, 3, and 4 alleles; DR2h: 0, 2, 3, 4, 5, and 6 alleles), and the quantitative trait using the Spearman's correlation test. Alleles with at least one pFDR value ≤ 0.001 are indicated as full circles and those with a least one p value 0.01≤p < 0.001 are reported as an empty circles (A and B). Dark vertical lines represent p value thresholds at -LOG10 = 2 (p = 0.01) and -LOG10 = 3 (p = 0.001). C3: complement factor 3; C4: complement factor 4; anti-dsDNA: anti-double stranded DNA antibodies; anti-Sm: anti-Smith antibodies; anti-RNP: anti-ribonucleopeptide antibodies; Hb: hemoglobin; WBC: white blood cell count; Plt: platelet count; ESR: erythrocyte sedimentation rate.*

Using a Spearman's rho pFDR value of 0.001 as threshold, a negative correlation was identified between complement C4 protein levels and HLA-C∗07:01 at the allele level (p = 0.001), and HLA-DR3h at the haplotype level (p = 2.7 × 10^−5^) (Fig. 3). This observation is in agreement with previous reports describing an over-representation of the *C4B* null allele, located in the HLA class III region, with the ancestral HLA-DR3h that includes HLA-C∗07:01 [39]. Although the *C4B* gene was not included here, this hypothesis is strengthened by two arguments: i) we identified no direct involvement of HLA-DR3h corresponding HLA proteins HLA-C∗07:01P, HLA-B∗08:02P, and HLA-DRB1∗03:01P, and ii) the association between HLA-DR3h and C4 protein levels was limited to ancestral HLA-DR3h (X-B∗08:01-X-DRB1∗03:01) as compared to the HLA-DR3h variant associated with Asian ancestry (HLA-C∗07:02∼HLA-B∗08:02∼rs9332730∼DRB1∗03:01). Moreover, an association between HLA-A∗03:01P, including HLA-A∗03:01 (03:01:01:01, and 03:01:27 but not 03:01:01:02N) and HLA-A∗03:20 alleles, and low white cell counts in jSLE was seen (p = 0.0007); a lower significance was reported for the HLA-A∗03:01 allele (p = 0.0015).

**Fig. 4:**
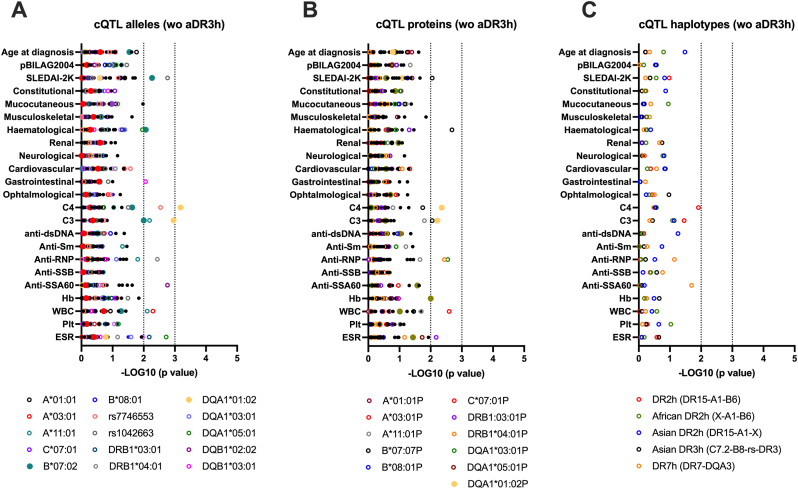
**jSLE-associated HLA quantitative trait loci after excluding individuals harboring the ancestral DR3 haplotype (B∗08:01-X-DRB1∗03:01). A**: Manhattan plot of HLA alleles (x A∗, x C∗, B∗, x C2-factor B, x DRB1∗, x DQA1∗, and xDQB1∗) and their association with jSLE features. **B:** Concordance with HLA protein sequence. **C:** Concordance with haplotypes HLA-DR2, -DR3, -DR7 and ancestral variants. On the x axis, –Log10(p values) are presented, assessing the concordance between allele/protein number (0, 1, and 2) or haplotype (HLA-DR3/DR7h: 0, 2, 3, and 4 alleles; HLA-DR2h: 0, 2, 3, 4, 5, and 6 alleles) and quantitative traits using the Spearman's correlation test. Alleles with at least one pFDR value ≤ 0.001 are indicated as full circles and those with a least one p value 0.01≤p < 0.001 are reported as an empty circles (A, B). Dark vertical lines represent p value thresholds at -LOG10 = 2 (p = 0.01) and -LOG10 = 3 (p = 0.001). C3: complement factor 3; C4: complement factor 4; anti-dsDNA: anti-double stranded DNA antibodies; anti-Sm: anti-Smith antibodies; anti-RNP: anti-ribonucleopeptide antibodies; Hb: hemoglobin; WBC: white blood cell count; Plt: platelet count; ESR: erythrocyte sedimentation rate.

Finally, the ancestral DR3h (B∗08:01-X-DRB1∗03:01) exerts a dominant effect. QTL analysis was repeated and confirmed a negative correlation between HLA-DQA1∗01:02 (but not DR2h) and C4 protein level after ancestral DR3h was excluded from the analysis (p = 0.0006) (Fig. 4).

## 4 Discussion

This study provides the first comprehensive mapping exercise of the HLA super-locus in jSLE across ancestral groups. It confirms previous observation in adult-onset SLE cohorts reporting prominent roles for DR2h and DR3h as risk factors. This underscores the importance of HLA super locus variation in the pathophysiology of SLE, including juvenile-onset disease. It also emphasizes the importance of ancestry-linked genetic diversity and its contribution to differential disease presentation and outcomes, especially affecting HLA-DR2h, -DR3h (associated SLE risk) and -DR7h (protective).

Associations between HLA-DR2h and disease risk has previously been reported in adult-SLE cohorts [40], and two possible mechanisms have been suggested. One relates to the presence of SLE-associated SNPs in strong linkage disequilibrium with HLA-DRB1∗15:01 present in the intergenic *XL9* region, located between HLA-DRB1 and HLA-DQA1, resulting in increased surface expression of these genes in HLA-DR2h carriers [41,42]. The second hypothesis, limited to HLA-DRB1∗15:01 in Asians, links a low copy number of the *C4A* gene with DR2h [43,44] (below). This would also explain the herein reported association between the DR2h-associated jSLE factor HLA-DQA1∗01:02 and a low C4 levels in Asian participants (after excluding DR3h). Across ancestral groups in this jSLE cohort, HLA-DQA∗01:02 was the most conserved DR2h-risk factor when comparing Caucasian (HLA-DRB1∗15:01∼DQA∗01:02∼DQB1∗06:02), Asian (HLA-DRB1∗15:01∼DQA∗01:02∼X), and African/Caribbean (X ∼ DQA∗01:02∼DQB1∗06:02) sub-cohorts.

In line with the identified negative association between DR7h and SLE in Asian participants in the current study, a protective role of DR7h in SLE has also previously been reported [45,46]. While associations between clinical phenotypes in DR7h were not seen in this cohort, others reported associations with predominantly cutaneous presentation [46,47], and specific autoantibody patterns including anti-Smith (Sm), anti-SSA/Ro, and anti-cardiolipin autoantibody positivity in adult-onset SLE cohorts [[48], [49], [50]]. Differences may be partially linked to the more severe phenotypes observed in juvenile-when compared to adult-onset SLE [1,4,7].

Associations between HLA variation and clinical characteristics exist and are complex, involving *HLA* alleles (HLA-DQA1∗01:02 and low C4 levels), HLA proteins (HLA-A∗03:01P and low white blood cell count), and the ancestral DR3h (with low C4 levels).

In jSLE, low C4 protein levels may either be the result of genetic factors or the consumption of the classical complement pathway (also associated with low C3) [51]. The link between a homozygous C4 deficiency affecting both *C4A* and *C4B* genes at the *HLA* class III region is extremely rare, unlike the presence of a *C4B* null allele leading to partial C4 deficiency previously reported in Caucasian adult-onset SLE patients carrying DR3h [52]. Furthermore, linkage disequilibrium between the *C4B* null allele (prevalence: 45–66 %) and DR3h (prevalence: 22–29 %) is partial in this ancestral group [39]. When *C4B* is duplicated, it associates with the presence of the minor allele *C2* SNP rs9332730-C [53]. The presence of rs9332730-C in the alternative Asian DR3h supports *C4B* gene duplication, which may explain normal C4 levels associated with the alternative DR3h. However, in the current study, we could not exclude underlying mechanisms other than C4, as both ancestral and Asian alternative DR3h were identified as risk factors for jSLE. Lastly, the recent identification of an epitope encoded HLA-DRB1∗03:01 that requires interferon-gamma to be functional suggests additional pathomechanisms, independent from antigen presentation to be involved in haplotype:phenotype associations [38].

The association between HLA-A∗03:01P, and to a lower extent also for HLA-A∗03:01, and low white cell counts points to a potential direct effect, that needs to be confirmed. The HLA-A∗03:01P allele has previously been associated with other immune-mediated diseases such as multiple sclerosis (MS) and type 1 diabetes [54,55]. In humanized mice, HLA-A∗03:01P contributes to the induction of MS after immunization with the myelin proteolipid protein-derived PLP45–53 peptide (KLIETYFSK) [56].

While this study provides the most comprehensive HLA study in jSLE to date, it has several limitations. Though a national cohort from the UK, the study population was relatively small, especially when considering individual ancestral groups. While here, SLE risk was tested at the “continental” ancestry level and compared to publicly available datasets, larger international cohorts may allow testing at “country level” which may allow for validation of minor associations and better characterization of SLE-associated haplotypes. With the notable exception of the *C2* region, non-HLA genes were not considered in the extended haplotype characterization, which may introduce a bias, as revealed with *C2* rs9332730 to differentiate Caucasian from Asian HLA-DR3h. Lastly, because of incomplete sequencing data, the *C4A* and *C4B* genes have not been included in this study. Thus, we cannot completely exclude that the association with C4 deficiency may result from activation of the classical complement pathway. However, this risk is limited as no association with low C3 levels was found.

In conclusion, this study underscores the importance of HLA variation as a risk factor in SLE, including jSLE. It promises potential for future patient stratification and risk assessment based on genetic profiling and underscores the importance of considering inter-ancestral differences in genetic composition and associated disease risk. Because jSLE frequently presents with “poly-autoimmunity”, findings from this study may also inform associated autoimmune/inflammatory diseases.

## CRediT authorship contribution statement

**Yves Renaudineau:** Writing – review & editing, Writing – original draft, Visualization, Validation, Supervision, Software, Resources, Methodology, Investigation, Formal analysis, Data curation. **Amandine Charras:** Writing – review & editing, Writing – original draft, Supervision, Software, Resources, Project administration, Methodology, Investigation, Formal analysis, Data curation. **Valentina Natoli:** Writing – review & editing, Validation, Resources, Project administration, Methodology, Investigation, Formal analysis, Data curation. **Nicolas Congy-Jolivet:** Writing – review & editing, Software, Methodology, Investigation, Formal analysis. **Sam Haldenby:** Writing – review & editing, Methodology, Investigation, Formal analysis, Data curation. **Xuan Liu:** Writing – review & editing, Methodology, Investigation, Formal analysis, Data curation. **Yongxiang Fang:** Writing – review & editing, Methodology, Investigation, Formal analysis, Data curation. **Eve MD. Smith:** Writing – review & editing, Resources, Methodology, Investigation, Data curation. **Michael W. Beresford:** Writing – review & editing, Resources, Project administration, Funding acquisition, Data curation, Conceptualization. **Christian M. Hedrich:** Writing – review & editing, Writing – original draft, Visualization, Validation, Supervision, Software, Resources, Project administration, Methodology, Investigation, Funding acquisition, Formal analysis, Data curation, Conceptualization.

## Declaration of competing interest

The authors report no conflict of interest.

## Data Availability

Data will be made available on request.
